# Towards sustainable funding for Recovery Colleges: lessons from Germany for advancing recovery-oriented psychiatry

**DOI:** 10.3389/fpsyt.2026.1903764

**Published:** 2026-10-01

**Authors:** Simon A. Stiehl, Jared Omundo, Nora Ambord, Christian Burr, Michael Schulz, Orkan Okan

**Affiliations:** 1 Medizinische Hochschule Brandenburg Theodor Fontane, Neuruppin, Germany; 2 Hochschule Osnabruck, Osnabrück, Germany; 3 Recovery College Osnabrück (RCO), Osnabrück, Germany; 4 Technische Universitat Munchen Department Health and Sport Sciences, Munich, Germany; 5 Recovery College Gütersloh-Ostwestfalen (GT-OWL), Gürtersloh, Germany; 6 Berner Fachhochschule, Bern, Switzerland; 7 Recovery College Bern, Bern, Switzerland; 8 Universitare Psychiatrische Dienste Bern, Bern, Switzerland; 9 LWL-Klinikum Gutersloh, Gürtersloh, Germany; 10 Technical University of Munich, Munich, Germany; 11 World Health Organization (WHO) Collaborating Centre for Health Literacy at Technical University Munich (TUM), Munich, Germany

**Keywords:** empowerment, funding, Germany, mental health, mental health literacy (MHL), personal recovery, Recovery Colleges

## Abstract

Recovery Colleges (RCs) are community-based, co-produced educational initiatives that promote personal recovery, mental health, and well-being through collaborative learning grounded in recovery principles and adult education. Rather than functioning as clinical interventions, Recovery Colleges provide educational environments in which people with lived experience, professionals, relatives, and community members engage in shared learning and knowledge exchange. Despite their growing international implementation, many Recovery Colleges continue to rely on insecure, short-term funding arrangements, limiting their long-term sustainability and integration within recovery-oriented mental health systems. This Perspective examines the rationale for sustainable structural support by synthesizing current conceptual developments, emerging empirical findings, and practice-based experiences, with particular attention to lessons from German-speaking countries. Five evidence-informed arguments are discussed across individual, organizational, system, and community levels: Recovery Colleges may contribute to (1) strengthening self-determination and empowerment, (2) complement recovery-oriented mental health systems through education and peer-supported learning, (3) contribute to reducing stigma and strengthening mental health literacy, (4) foster co-production and recovery-oriented organizational cultures, and (5) support social inclusion and community participation. Although qualitative and mixed-methods studies increasingly suggest beneficial experiences associated with Recovery College participation, the current evidence base remains heterogeneous, and conclusions regarding effectiveness, cost-effectiveness, and broader system-level impacts require further high-quality research. Accordingly, Recovery Colleges should be understood as promising recovery-oriented educational infrastructures rather than established clinical interventions with demonstrated effectiveness. Sustainable structural support may enable Recovery Colleges to maintain their educational philosophy, co-production principles, and community-based orientation while becoming more sustainably embedded within mental health systems. Investment in Recovery Colleges therefore represents an investment in participatory, recovery-oriented infrastructures that recognize lived experience as a valuable source of knowledge and strengthen opportunities for inclusion, empowerment, and collaborative learning.

## 1 Introduction and background

Recovery has become a central paradigm in contemporary mental health care, shifting the focus beyond symptom reduction towards supporting individuals to live meaningful, self-determined lives despite mental health challenges (1–3). This recovery-oriented approach is increasingly reflected in international policy frameworks including the World Health Organization’s Comprehensive Mental Health Action Plan 2013–2030 (4), which emphasizes person-centered, rights-based, and community-oriented mental health systems. Recovery is understood not merely as a clinical outcome but as a deeply personal and social process involving hope, identity, connectedness, meaning, and empowerment (5, 6). Importantly, contemporary recovery-oriented approaches extend beyond the delivery of individual clinical interventions by promoting participation, autonomy, social inclusion, and recognition of lived experience as a source of expertise. This broader perspective has stimulated the development of educational and community-based approaches that complement existing mental health services through collaborative learning, empowerment and participation.

### 1.1 Recovery Colleges as recovery-oriented educational infrastructures

Recovery Colleges (RCs) emerged from the convergence of the international recovery movement and traditions of adult education. Their origins can be traced to Recovery Education Centers (RECs) established in the United States during the 1990s through collaborations between people with lived experience and adult education providers (3). The first formal Recovery College was subsequently established in England in 2009, further developing the Recovery Education Center model within a recovery-oriented educational framework (3, 7, 8). Rather than representing an alternative form of treatment, RCs are educational environments in which people with lived experience, family members, professionals, and community members engage in shared learning.

Grounded in principles of co-production, experiential knowledge, and adult learning, Recovery Colleges recognize lived experience as a legitimate source of expertise alongside professional and scientific knowledge (9–11). Instead of relying on traditional expert-led models, they promote dialog, mutual learning, and shared decision-making in ways that seek to reduce power differentials and foster self-determination (12–14). Their primary purpose is not diagnosis or treatment, but supporting personal development, recovery, and participation through learning. Consistent with adult education principles, participants are regarded as experts in their own lives and are supported in developing knowledge, confidence, and skills relevant to their individual recovery journeys (8, 15, 16).

### 1.2 International development and diversity of implementation

Over the past decade, Recovery Colleges have expanded considerably. A recent international mapping study identified 221 Recovery Colleges operating across 28 countries on five continents, reflecting both the global uptake of the model and its adaptation to diverse cultural and health system contexts (17). Despite this expansion, Recovery Colleges do not represent a standardised intervention. Instead, they are adapted to diverse healthcare systems, community structures, organizational cultures, and available resources, resulting in considerable variation in governance, funding arrangements, course content, and implementation strategies (18–21). This diversity highlights the flexibility of the model while illustrating that Recovery Colleges are shaped by local needs and sociocultural contexts rather than following a single implementation approach.Within German-speaking countries, Recovery Colleges, Empowerment Colleges (which apply similar educational and co-produced principles under a different name), and more recently Discovery Colleges (which extend these principles to children and adolescents), have become increasingly established. Collaborative network has also emerged to promote mutual learning quality development and exchange of implementation experiences (22–26). These developments suggest that Recovery Colleges operate across multiple interconnected levels: they influence individual experiences, shape organizational cultures and professional practice, interact with broader mental health systems, and contribute to community participation and inclusion.

### 1.3 Sustainable support as a key challenge

Despite increasing international implementation, securing sustainable funding remains an important challenge for many Recovery Colleges. In numerous countries, including Germany, RCs continue to depend on temporary project funding, charitable support, or local initiatives, creating uncertainty regarding their long-term sustainability despite increasing policy interest in recovery-oriented mental health care. For example, in Germany, the 2025 Psychiatry Plan of the federal state of North Rhine-Westphalia explicitly highlights the importance of Recovery Colleges and calls for more sustainable institutional and financial support (27). At the same time, discussions regarding sustainability should recognize that the distinctive contribution of Recovery Colleges lies not only in their existence as educational programs, but also in preserving the principles that define them: co-production, experiential knowledge, relative independence from clinical structures, and community accessibility (24).

### 1.4 Aim of this perspective

Against this background, this Perspective synthesizes current conceptual developments, emerging empirical evidence, and practice-based experiences to examine arguments supporting sustainable structural support for Recovery Colleges and related educational initiatives, including Empowerment Colleges and Discovery Colleges. Rather than presenting Recovery Colleges as replacements for existing mental health services or as interventions with established effectiveness, we argue that they can be understood as promising recovery-oriented educational infrastructures whose long-term contribution depends on sustainable implementation while maintaining their foundational values. To structure the discussion, the manuscript adopts a multilevel perspective comprising four interconnected levels. It first considers the potential contribution of Recovery Colleges to individual recovery and empowerment, before examining their organizational characteristics, their positioning within wider mental health systems, and their role in promoting community participation and inclusion. This structure provides a coherent framework for discussing the opportunities, challenges, and current evidence relating to sustainable structural support for Recovery Colleges.

## 2 Multilevel arguments for sustainable structural support of Recovery Colleges

Building on the conceptual framework introduced abovethe following sections examine the potential contribution of Recovery Colleges across four interconnected levels: individual, organizational, system, and community. This multilevel perspective provides a structured framework for discussing the conceptual foundations, emerging empirical evidence, and practical considerations relevant to sustainable structural support (see **Figure 1**). At the individual level, the discussion considers how Recovery Colleges may contribute to personal recovery, empowerment, and self-determination. The organizational level focuses on co-production, experiential knowledge, and the development of recovery-oriented organizational cultures. The system level examines the role of Recovery Colleges as complementary educational infrastructures within contemporary mental health systems. Finally, the community level considers their potential contribution to mental health literacy, social inclusion, and community participation. Although these levels are presented separately to facilitate discussion, they are closely interconnected and mutually reinforcing. Recovery Colleges operate across these different levels simultaneously, and their potential contribution emerges through the interaction of individual, organizational, system, and community processes. The following sections therefore discuss each level in relation to the available conceptual and empirical evidence while acknowledging the strengths, limitations, and remaining gaps in the current knowledge base (28).

**Figure 1:**
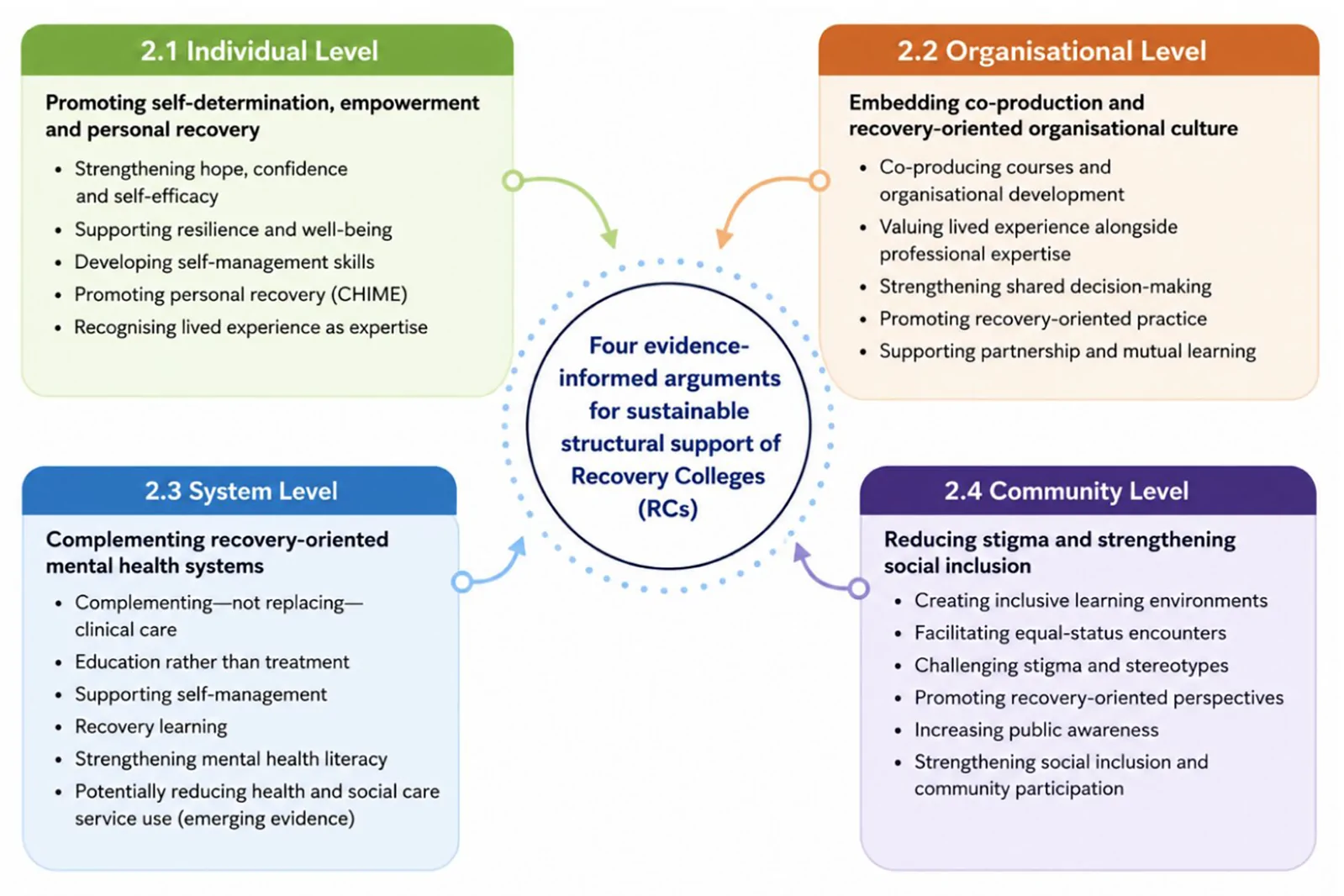
Multilevel framework for discussing sustainable structural support for Recovery Colleges (authors’ illustration).

### 2.1 Individual level: promoting self-determination, empowerment and personal recovery

Promoting self-determination and empowerment represents one of the central principles of the recovery movement and a defining characteristic of Recovery Colleges (1, 3, 10). As co-produced educational environments, RCs encourage participants to engage as active learners and to recognize lived experience as a valuable source of knowledge alongside professional expertise. Through collaborating learning with peers, family members, professionals, and community members, participants have opportunities to develop knowledge, confidence, and practical skills that support their individual recovery journeys (11, 12, 14). This educational approach aligns with the CHIME framework of personal recovery, which conceptualizes recovery in terms of connectedness, hope and optimism, identity, meaning in Life, and empowerment (5). By creating opportunities for reflection and shared learning, Recovery Colleges may support individuals in strengthening their sense of agency, recognizing personal strengths, and developing recovery-oriented skills. Emerging qualitative, quantitative, and mixed-methods research suggests that participation in Recovery Colleges may be associated with improvements in several dimensions of personal recovery, including empowerment, hope, confidence, self-management, and social connectedness. (13, 29–31).

Recent evidence syntheses likewise suggest that Recovery College participation is associated with positive recovery-related experiences, while highlighting considerable methodological heterogeneity and the need for further controlled, longitudinal, and comparative research (32–34). Taken together, the available evidence suggests that Recovery Colleges represent promising educational environments that may support empowerment and self-determination. However, the current evidence remains insufficient to draw conclusions regarding effectiveness, and further research is required to clarify mechanisms of action, contextual influences and long-term outcomes.

### 2.2 Organizational level: embedding co-production and recovery-oriented organizational cultures

Co-production represents a defining organizational principle of Recovery Colleges (RCs), shaping not only educational activities but also governance, organizational development, and decision-making processes. Rather than limiting the involvement of people with lived experience to consultation or feedback, Recovery Colleges seek to create organizational structures in which lived experience, professional knowledge, and educational expertise contribute on an equal footing to planning, delivery, evaluation, and strategic development (9, 10, 12). This organizational approach creates opportunities for people with lived experience to participate not only as learners but also as peer educators, course developers, advisory board members, and organizational partners, thereby extending participation beyond traditional models of service-user involvement (13, 14). Recovery Colleges have adopted diverse organizational arrangements, including independent community-based initiatives, peer-led organizations, jointly governed models, and programs associated with established mental health services (24, 35). Although the extent and form of lived-experience involvement varies across settings, co-production remains a common organizational principle underpinning many Recovery College implementations (11).

Emerging evidence suggests that co-production within Recovery Colleges may contribute to more recovery-oriented organizational cultures. Studies suggest that professionals, involved in Recovery Colleges report greater appreciation of experiential knowledge, increased confidence in recovery-oriented practice, and more collaborative relationships with people using mental health services (29, 36–38). At the organizational level, these developments may support cultures characterized by partnership, shared decision-making, and recognition of multiple forms of expertise. However, these organizational changes should not be interpreted as automatic consequences of establishing a Recovery College. Meaningful co-production requires sustained organizational commitment, appropriate resources, supportive governance structures, and opportunities for genuine participation rather than symbolic involvement. This is particularly relevant as Recovery Colleges become increasingly integrated into formal mental health systems. While institutional support may strengthen accessibility and long-term sustainability, increasing institutionalisation may also create tensions with core Recovery College principles, including flexibility, shared ownership, and the relatively equal relationships between participants and professionals. Sustainable structural support should therefore seek not only ensure financial stability but also preserve the organizational conditions that enable authentic and recovery-oriented practice.

### 2.3 System level: complementing recovery-oriented mental health systems

Recovery Colleges complement recovery-oriented mental health systems by providing educational opportunities alongside, rather than in place of, existing mental health services. Their primary contribution lies expanding the range of recovery-oriented supports available to individuals by creating spaces for learning, self-management, participation, and collaborative knowledge exchange. Positioned at the interface of healthcare, education, and community development, Recovery Colleges represent a complementary component of broader mental health systems rather than an alternative model of clinical care (3, 11, 16). From a system perspective, Recovery Colleges may strengthen recovery-oriented care by broadening opportunities for participation, supporting collaboration across sectors, and fostering partnerships between healthcare providers, educational institutions, municipalities, and community organizations.

Emerging evidence suggests that participation in Recovery Colleges may be associated with changes in patterns of health and social care utilization, including reductions in some forms of service use among certain participant groups (39, 40). However, these findings should be interpreted cautiously given substantial variation in participant characteristics, local service structures, study designs, and evaluation methods. Similarly, the current evidence regarding economic outcomes remains limited and methodologically heterogeneous (32). Consequently, the value of Recovery Colleges should not be assessed primarily through healthcare utilization or economic outcomes alone. Importantly, the value of Recovery Colleges should not be assessed solely in terms of healthcare utilization or economic outcomes. Rather, evaluations should consider whether Recovery Colleges support people in accessing forms of learning and support that are meaningful, timely, and aligned with their individual recovery goals and preferences. From a ssustainability perspective structural support may enable Recovery Colleges to establish stable organizational infrastructures and long-term cross-sector partnerships while maintaining the educational orientation, co-production, and relative independence that distinguish Recovery Colleges from conventional clinical services.

### 2.4 Community level: strengthening social inclusion, mental health literacy and community participation

Recovery Colleges extend beyond individual learning by creating community-based educational environments in which people with lived experience, relatives, professionals, and members of the wider public participate as learners on an equal footing. By bringing together individuals with diverse backgrounds and experiences, they create opportunities for dialog, reciprocal learning, and relationship-building that extend beyond traditional mental health service boundaries (12, 16, 41). In doing so, Recovery Colleges may help foster more collaborative relationships and strengthen connections between people who might otherwise have limited opportunities for meaningful interaction. Qualitative studies consistently describe Recovery Colleges as respectful, inclusive, and psychologically safe learning environments in which experiential knowledge is recognized, and different perspectives are valued (13, 31, 38).

### 2.5 Mental health literacy and stigma

Beyond supporting individual learning, Recovery Colleges may also contribute to strengthening mental health literacy within communities. Through collaborative educational activities, participants are encouraged to discuss mental health, recovery, and lived experience in ways that may strengthen understanding, reduce stigma associated with mental health difficulties and help-seeking, and increase confidence in talking about mental health (29, 41, 42). These aims are conceptually consistent with contemporary mental health literacy frameworks (43), although empirical evidence examining literacy-related outcomes within Recovery Colleges remains comparatively limited (32)).

### 2.6 Social inclusion and community participation

Recovery Colleges may also promote social inclusion by creating opportunities for people to participate in education, volunteering, employment, peer support, advocacy, and wider community activities (11, 14). Consistent with contemporary recovery theory, recovery is understood not only as an individual process but also as one shaped by opportunities for participation, belonging, citizenship, and meaningful relationships (5, 6). Emerging qualitative and mixed-methods research suggests that participants often describe increased feelings of belonging, social connectedness, and opportunities for community participation, although these experiences vary across individuals and implementation contexts (13, 15, 31).

### 2.7 Recovery Colleges as community-bridging organizations

Recovery Colleges may further contribute to recovery-oriented communities by acting as community-bridging organizations that connect healthcare providers, educational institutions, municipalities, voluntary organizations, peer networks, and civil society. These partnerships have the potential to strengthen collaboration across sectors and support more coordinated, recovery-oriented approaches within local communities (17, 24, 25). However, these broader community-level contributions remain comparatively under-researched. While qualitative studies provide valuable insights into participants’ experiences, further implementation, longitudinal, and community-level research is required to better understand how Recovery Colleges influence mental health literacy, social inclusion, community participation, and public mental health over time. Taken together, current evidence suggests that Recovery Colleges have the potential to contribute to more inclusive and recovery-oriented communities by creating educational spaces that encourage participation, dialog, and mutual learning. Nevertheless, these community-level contributions should be regarded as promising developments rather than established outcomes, reflecting the evolving nature of the evidence base.

## 3 Conclusion

Recovery Colleges (RCs) represent a distinctiverecovery-oriented approach that combines adult learning, co-production, and lived-experience expertise within community-based settings. This Perspective has examined potential contribution across four interconnected levels: individual, organizational, system, and community, highlighting how Recovery Colleges may support personal recovery, foster recovery-oriented organizational cultures, complement contemporary mental health systems, and strengthen community participation and inclusion. At the same time, the available evidence should be interpreted with appropriate caution. Although the literature has grown substantially over the past decade, the evidence base remains methodologically heterogeneous and is dominated by qualitative, mixed-methods, and observational research. Consequently, current findings primarily provide evidence regarding participants’ experiences and perceived benefits, whereas robust conclusions regarding effectiveness, cost-effectiveness, and broader system-level impacts remain limited. Accordingly, sustainable structural support should not be justified solely on the basis of demonstrated effectiveness or anticipated economic benefits. Rather, the rationale for supporting Recovery Colleges lies in their potential to strengthen recovery-oriented educational infrastructures that promote co-production, participation, experiential knowledge, and collaborative learning. Any broader organizational or system-level benefits should currently be regarded as promising but still evolving areas of research.

Future research should therefore prioritize longitudinal, comparative, implementation, and economic evaluations alongside continued qualitative inquiry. Such research is needed to better understand not only whether Recovery Colleges are effective, but also how, for whom, and in which contexts they contribute to recovery-oriented mental health systems. Particular attention should be given to implementation processes, organizational sustainability, and community-level outcomes, which remain comparatively underexplored. Taken together, current conceptual developments and emerging empirical evidence suggest that Recovery Colleges represent a promising area for continued implementation, evaluation, and policy development. As their implementation expands, sustainable structural support should seek not only to ensure organizational stability but also to preserve the defining principles of Recovery Colleges, including co-production, lived-experience expertise, educational autonomy, and community accessibility. Continued research will be essential to strengthen the evidence base and clarify the longer-term contribution of Recovery Colleges within recovery-oriented mental health systems.

## Data Availability

The original contributions presented in the study are included in the article/**Supplementary Material**. Further inquiries can be directed to the corresponding authors.
