# Supplementary material for: Towards sustainable funding for Recovery Colleges: lessons from Germany for advancing recovery-oriented psychiatry
Source: Front Psychiatry. 2026 Oct 1;17:1903764. doi: 10.3389/fpsyt.2026.1903764 (PMC13669186; doi:10.3389/fpsyt.2026.1903764)
Supplement: Supplementary file 1 [file SupplementaryFile1.docx]

**Supplementary Appendix 1**

**Illustrative examples of Recovery College implementation models in German-speaking countries**

**Purpose of this appendix**

This appendix provides three illustrative examples of Recovery College implementation in German-speaking countries. The examples were selected to demonstrate variation in organisational structures, governance arrangements, funding models, and implementation contexts. They are not intended to represent the full diversity of Recovery College models or to provide evaluations of effectiveness. Rather, they illustrate how recovery-oriented educational approaches have been adapted across different regional and organisational settings.

**Supplementary Table S1.** Illustrative examples of Recovery College implementation models in German-speaking countries (Stiehl et al., 2024)

| **Recovery College** | **Country** | **Established** | **Organisational model** | **Funding** | **Key implementation characteristics** |
| --- | --- | --- | --- | --- | --- |
| Recovery College Osnabrück (RCO) | Germany | 2019 | Independent community-based initiative | Project-based funding and donations | Co-produced courses, volunteer involvement, collaboration with universities and community organisations |
| Recovery College Gütersloh–Ostwestfalen (RC GT-OWL) | Germany | 2018 | Association in cooperation with psychiatric services | Foundation support and institutional funding | Focus on mental health literacy, EX-IN education, and recovery-oriented organisational development |
| Recovery College Bern (RCB) | Switzerland | 2019 | Independent association with public service agreement | Longer-term public funding agreement with the Canton and City of Bern | First Recovery College in German-speaking Switzerland; publicly supported model with regional expansion |

**Recovery College Osnabrück (RCO), Germany**

Recovery College Osnabrück (RCO) is a community-based educational initiative focusing on mental health, personal recovery, and psychosocial well-being. Established in 2019, the initiative emerged from collaboration between the self-help organisation Psychiatrieerfahrene/Trialog Osnabrück e. V., university students, the community association Weitblick e. V., and established Recovery Colleges, including Recovery College Bern and Recovery College Gütersloh (Stiehl 2022). The organisational development was supported by a collaborative working group (AG Recovery College Osnabrück), which contributed to the development of the college structure, educational approach, and course programme between 2019 and 2022. Initial implementation was supported through a 5,000 euro award from the Theodor Fliedner Prize. RCO follows the principles of recovery, adult education, and co-production. Courses are jointly developed and delivered by people with lived experience and professionals and address topics including hope, identity, self-care, crisis management, and coping with psychological distress (Stiehl 2022; Stiehl et al. 2024). The initiative operates as an independent community-based model and collaborates with volunteers, people with lived experience, professionals, and educational partners. Courses are offered free of charge or through voluntary donations. RCO is currently hosted by the socio-cultural organisation K.A.F.F. e. V. in Osnabrück and operates independently of formal psychiatric care structures.

Further information and the current course programme are available at:
<https://weitblicker.org/media/documents/osnabrueck/recovery_kursangebot_sommer_2026.pdf>

**Recovery College Gütersloh–Ostwestfalen (RC GT-OWL), Germany**

Recovery College Gütersloh–Ostwestfalen (RC GT-OWL) is a community-based educational initiative established in 2018 that provides courses, workshops, and seminars related to mental health literacy, recovery, and well-being. The initiative was developed through collaboration between people with lived experience, mental health professionals, and community members. Its activities include accessible educational offers for the public, opportunities for social connection, recovery-oriented training for professionals and organisations, and peer support activities, including EX-IN qualification programmes. In accordance with Recovery College principles, courses are co-developed and co-facilitated by people with lived experience and professionals from different disciplines, including psychiatry, nursing, psychology, social work, and occupational therapy. RC GT-OWL is operated by ReOrGe e. V. in cooperation with LWL Clinic Gütersloh and receives financial support from the LWL Social Foundation and the Gütersloh Citizens’ Foundation. The initiative offers approximately 40–50 seminars and workshops per semester and has engaged more than 1,000 participants since its establishment (Omundo et al. 2025).

Further information is available at: <https://www.rcgt-owl.de/>

**Recovery College Bern (RCB), Switzerland**

Recovery College Bern (RCB) was founded in 2019 as the first Recovery College in the German-speaking part of Switzerland. Initially established as a project of the University Psychiatric Services Bern (UPD), it later became part of the association IG Sozialpsychiatrie Bern (igs Bern) and developed additional organisational support structures. Following financial challenges in 2024, stakeholders involved with the RCB engaged in discussions regarding long-term sustainability. Since 2025, the RCB has been supported through a longer-term service agreement with the Canton and City of Bern. The RCB offers approximately 35 co-facilitated courses per semester and involves people with lived experience, professionals, and community members as learners and course facilitators. Courses are delivered according to the principles of co-production and shared learning, with participation fees adjusted according to income. The RCB represents an example of a publicly supported Recovery College model and is currently extending its activities to additional locations within the canton.

Further information is available at: <https://www.recoverycollegebern.ch>

**Summary**

These three examples illustrate different approaches to Recovery College implementation in German-speaking countries, including community-based, service-affiliated, and publicly supported organisational models. Despite differences in governance, funding arrangements, and regional contexts, all three initiatives share core Recovery College principles, including co-production, adult education, and recognition of lived experience as a source of expertise. Together, these examples provide practical illustrations of the implementation diversity discussed in the main manuscript and highlight that sustainable support may require context-sensitive approaches rather than a single organisational model.

**References**

Omundo, Jared, Simon A. Stiehl, Michael Schulz, Andrea Zingsheim, Susanne Schmedthenke, Michael Löhr, and Klaus-Thomas Kronmüller. 2025. “Examining Recovery College Experiences: Perspectives on Learning and Personal Growth From an Online Survey.” *Journal of Psychosocial Rehabilitation and Mental Health* 13 (2): 215–25. https://doi.org/10.1007/s40737-024-00448-6.

Stiehl, Simon. 2022. “Recovery-College: Hochschule für psychische Gesundheit.” *PPH* 28 (1): 18–24. https://doi.org/10.1055/a-1638-3722.

Stiehl, Simon, Jared Omundo, Klaus-Thomas Kronmüller, et al. 2024. “Förderung und Prävention von seelischer Gesundheit und Wohlbefinden in der Gemeinde.” *Prävention und Gesundheitsförderung* 19: 528–36. https://doi.org/10.1007/s11553-023-01075-y.
